# A randomized, double-blind study on the efficacy of oral domperidone versus placebo for reducing SARS-CoV-2 viral load in mild-to-moderate COVID-19 patients in primary health care

**DOI:** 10.1080/07853890.2023.2268535

**Published:** 2023-10-17

**Authors:** Alejandro Rabanal Basalo, Mercedes Navarro Pablos, Nuria Viejo Pinero, María Luz Vila Méndez, Verónica Molina Barcena, Aránzazu Montilla Bernabé, María del Pilar Villanueva Morán, Ana María Blanco Gallego, Carmen Guirao Sánchez, Salvador Juárez Antón, Ángela Fernández Rodríguez, María Luisa Revuelta Puigdollers, María Teresa Sarriá Sánchez, Carmen Martín Alegre, Miguel Ángel Martínez Álvarez, María Mestre de Juan, Rebeca Mielgo Salvador, María Teresa Gijón Seco, José Manuel Saníger Herrera, María Esther Rodríguez Jiménez, Begoña Navas de la Peña, Javier Santa Cruz Hernández, Ana María Abad Esteban, Rebeca Díaz Martín, Laura García Pérez, Paloma Herrero Vanrell, María Isabel Arias de Saavedra Criado, Alexandra Vaquero Vinent, Verónica López Gómez, Víctor Manuel Montegrifo Rentero, Lucía Simón Miguel, Ignacio Campo Martos, Silvia Ortiz Zamorano, María Jesús Izquierdo Zamarriego, Izíar Vázquez Carrión, Rosa María López Valero, Carmen Gil, Ana Martínez, Begoña Soler López

**Affiliations:** a C.S. Los Yébenes, Madrid, Spain; b C.S. Fronteras, Torrejón de Ardoz, Spain; c C.S. Benita de Ávila, Madrid, Spain; d C.S. Baviera, Madrid, Spain; e C.S. Los Alperchines, San Fernando de Henares, Spain; f C.S. General Fanjul, Madrid, Spain; g C.S. Daroca, Madrid, Spain; h C.S. Reina Victoria, Madrid, Spain; i Clínica Monmar, Las Rozas, Spain; j C.S. Las Rozas El Abajón, Las Rozas, Spain; k C.S. Villablanca, Madrid, Spain; l C.S. Dos de Mayo, Móstoles, Spain; m Centro de Investigaciones Biológicas ‘Margarita Salas’, CSIC, Madrid, Spain; n Centro de Investigación Biomédica en Red en Enfermedades Neurodegenerativas, ISCiii, Madrid, Spain; o Medical Department, E-C-BIO, S.L., Las Rozas, Spain

**Keywords:** SARS-CoV-2 virus, COVID-19 disease, domperidone, PCR

## Abstract

**Introduction:**

The clinical effect of domperidone against COVID-19 has been investigated in a double-blind phase III clinical trial (EudraCT number 2021-001228-17). Domperidone has shown in vitro antiviral activity against severe acute respiratory syndrome coronavirus 2 (SARS-CoV-2) and potential immudolatory properties through the stimulation of prolactin secretion.

**Patients and methods:**

The efficacy of oral domperidone plus standard of care (SOC; *n* = 87) versus placebo plus SOC (*n* = 86) was evaluated in a 28-day randomized double-blind multicentre study in primary health care centres. A total of 173 outpatients with mild-to-moderate COVID-19 were included. Three daily doses of 10 mg (30 mg/day) of domperidone or placebo were administered for 7 days. Reduction of viral load on day 4 was the primary efficay endpoint. It was estimated in saliva samples by reverse transcription-quantitative polymerase chain reaction (RT-qPCR), as the cycle thresholds detected ORF1ab, N Protein and S Protein genes.

**Results:**

A significant reduction in the viral load was observed (*p* < 0.001) from baseline to days 4, 7 and 14 of the three genes studied with non-significant differences between domperidone and placebo groups. Twenty-three patients (13.3%) experienced adverse events, 14 patients in the domperidone group (16.1%) and 9 patients in the placebo group (10.5%). No patients needed to be hospitalized.

**Conclusion:**

Results do not prove the use of domperidone as antiviral in patients with COVID-19.

## KEY MESSAGES

- A 28-day double-blind clinical trial was performed to investigate the antiviral effect of domperidone, 30 mg/day for 7 days (*n* = 87) versus placebo (*n* = 86) in outpatients with mild-to-moderate COVID-19.
- The primary efficacy endpoint was the reduction of viral load on day 4 as compared with baseline, estimated as the cycle thresholds to detect ORF1ab, N Protein and S Protein genes by RT-qPCR in saliva samples.
- The study findings do not prove the use of domperidone as antiviral in patients with COVID-19.

## 1. Introduction

Domperidone is a dopaminergic antagonist with antiemetic properties, marketed with indication for the relief of symptoms of nausea and vomiting. It is well tolerated and safe but can produce increasement in the QT interval, the time from the start of the Q wave to the end of the T wave ortime taken for ventricular depolarisation and repolarisation. Thus, it has warnings when used for patients with cardiac disease or with concomitant use of other drugs affecting the QT interval. This drug stimulates the secretion of prolactin in the pituitary gland as a secondary effect.

The clinical trial here reported explored the antiviral activity in clinic of domperidone, as a drug repositioning strategy. Although the cellular mechanism of action of domperidone as an antiviral is unknown, there are some published data on the potential role of domperidone for the management of COVID-19. Moreover, our research group found *in vitro* antiviral activity in different cell lines, including human lung cells Calu, that justified the initiation of the phase III, efficacy clinical trial [1,2]. Furthermore, stimulation of prolactin secretion in the pituitary gland using dopamine antagonists such as domperidone may enhance the protective effects of the immunomodulatory systems. This fact has been postulated as a medical hypothesis to assay this antiemetic agent as an anti-COVID-19 drug [3]. Prolactin has been associated with more than 300 different biological functions. It is involved in neuroendocrine, immune and haematopoietic regulation in conditions in which its functioning is altered. It occurs not only in the anterior part of the pituitary gland but also in other locations such as immune cells, neurons, prostate, decidua, mammary epithelium and skin [4,5]. As prolactin is also synthesized by lymphocytes, it is considered as a cytokine that acts on the same metabolic pathways as the cytokines of the immune system [5]. Prolactin plays a ­significant role in adaptive immunity, both humoral and cellular, through endocrine, paracrine and ­autocrine mechanisms [6–9]. Typically, prolactin elicits a ­proinflammatory response mediated by T helper lymphocytes type 1 (Th1) [10,11], and hyperprolactinemia is recognized as a pathological state (except during pregnancy) responsible for autoimmune diseases such as systemic lupus erythematosus, multiple sclerosis, rheumatoid arthritis, which are more prevalent in women, possibly because their prolactin levels are higher than in men [6,7,12–15].

Prolactin is involved in many other pathways that protect the immune system. It stimulates B and T cells, with a direct correlation between prolactin levels and the number of B and T lymphocytes [6,7]. A correlation between hyperprolactinemia and death in preterm newborns [16] has also been observed. In cases of trauma with severe bleeding, it has been observed that women with a higher level of prolactin have a longer survival than men, having proven that with the administration of metoclopramide (a dopamine antagonist that increases blood levels of prolactin) the immune system of patients affected by severe trauma can be stimulated favouring greater survival [17]. In patients with HIV, increasing blood prolactin by imipramine (dopamine antagonist) stimulates patients’ immunity by increasing CD4 cell count [18]. Stimulation of prolactin production has also been observed to reduce intervertebral disc degeneration, graft-versus-host disease, asthma and pulmonary allergic inflammatory response [9,19–21].

In addition to prolactin’s role in enhancing adaptive immunity, it has a key role in innate immunity, which is the first line of defense against pathogens, which stimulates NK cells, macrophages, neutrophils and dendritic cells [7,22,23]. It has been published that prolactin induces phagocytosis of *Candida albicans, Staphylococcus epidermidis, Staphylococcus aureus, Toxoplasma gondii* and *Acanthamoeba castellani* [22]. Elevated serum prolactin levels have also been shown to improve survival in patients with sepsis by activating the innate and adaptive immune system [24]. In fact, prolactin insufficiency has been documented in more than 50% of cases with neonatal sepsis [25].

From this rationale it follows that the controlled increase in blood prolactin levels at physiological levels or inducing mild hyperprolactinemia, using dopamine antagonists, could stimulate innate and adaptive immunity and increase the survival of patients in certain critical situations, among which can be found patients infected with SARS-CoV-2.

The present randomized double-blind clinical trial was conducted to assess the efficacy of domperidone to reduce the viral load in patients with mild-to-moderate COVID-19 in the primary healthcare setting.

## 2. Patients and methods

### 2.1. Study design and objectives

This was a phase III, randomized, double-blind, parallel group, placebo controlled, multicentre clinical trial conducted in 17 primary healthcare centres located in autonomous community of Madrid, Spain. The study period began on 22 March 2022 and finished on 3 November 2022. Each patient was followed up for 28 days.

The primary objective was to assess the efficacy of domperidone versus placebo on reducing viral load at day 4 from baseline. Secondary objectives included evaluating the efficacy of domperidone in achieving negative polymerase chain reaction (PCR) from baseline, to reduce the severity and duration of symptoms, to assess the need of medical care, admission to the hospital and oxygen therapy, and the mortality rate at day 28. All secondary objectives were included in the analysis to explore time and clinical consequences of exposure to viral load as the reduction in viral load is related to a lower frequency in the onset of symptoms, a lower severity of symptoms and a lower likelihood of hospital admission, as well as a reduction in the contagiousness of the virus.

The study was conducted in accordance with the last version of the Declaration of Helsinki and approved by the Medicinal Product Research Ethics Committee of Hospital Universitario Puerta de Hierro, Majadahonda (Madrid, Spain; code 1/2022, approval date 25 January 2022). The study was registered at European Union Drug Regulating Authorities Clinical Trials Database (EudraCT) with number 2021-001228-17. All the patients signed a written informed consent.

### 2.2. Patients

Eligible subjects were men or women aged 18 years or older, diagnosed with active SARS-CoV-2 infection confirmed by a positive rapid antigen detection test or a PCR test for viral RNA detection in the presence of compatible symptoms (fever, cough, shortness of breath or difficulty breathing, sore throat, body or muscle pain, fatigue, headache, chills, nasal congestion, loss of taste or smell, nausea or vomiting, and diarrhoea). It was required to have one or more symptoms within the last 72 h, with mild or moderate severity. Asymptomatic patients were eligible for the study if they had tested positive for SARS-CoV-2 within the previous 3 days. Exclusion criteria were patients living with a patient who had been enrolled in the present study, in order to avoid the exchange of medication between patients in the same household; patients with severe COVID-19; presence of diseases that may be affected or interfere with the results of the study (such as active infections other than SARS-CoV-2 requiring systemic therapy, uncontrolled respiratory disorder, prior ischemic heart disease, heart failure or atrial fibrillation, severe renal failure, active or treated malignancy, immunosuppression status, expected elective surgery within 30 days after screening for the study, severe obesity); concomitant treatment with drugs with known antiviral potential as remdesivir, lopinavir/ritonavir, chloroquine/hydroxychloroquine, tocilizumab, sarilumab, ruxolitinib, siltuximab, baricitinib, anakinra, interferon beta-1B, interferon alpha-2B, but not limited to this list; patients with domperidone use contraindications were excluded as patients with hypersensitivity or intolerance to domperidone or to any of the excipients, patients with prolactin-secreting pituitary tumour (prolactinoma), patients in whom stimulation of gastric motility may be dangerous, such as gastrointestinal bleeding, mechanical obstruction or perforation, patient with moderate-to-severe liver failure, with results in liver function tests, aspartate transaminase, alanine transaminase, alkaline phosphatase ≥5 times the upper limit of normal, patients with clinically significant abnormalities on the 12-lead electrocardiogram that may have been performed during the study screening period, specifically existing prolongation of cardiac conduction intervals, corrected QT interval, patient with underlying heart disease such as congestive heart failure or bradycardia, patient with significant electrolyte disorders such as hypokalaemia, hyperkalaemia, hypomagnesaemia, patient under treatment with drugs that prolong the QT interval, patient under treatment with potent CYP3A4 inhibitor drugs. Also, were excluded pregnant or breast-feeding women; patients unable to understand the informed consent; ineligibility as judged by the investigators; and participation in a clinical trial within the last 30 days. All the patients were informed about the study procedures and signed the informed consent form.

### 2.3. Randomization and intervention

Randomization was generated by an independent technician using a web-based randomization system (http://www.randomization.com; accessed on 20 November 2022). Patients were randomized 1:1 to the active treatment or the placebo arm according to an allocation sequence in random blocks of four and six treatments for a total of 10 treatments to be supplied to each study centre. After the patient signed the informed consent, the investigator assigned the consecutive blinded randomization number. This study had a double-blind design, so the physician, study personnel, the patients, the study monitors and the sponsor did not know if the patient was receiving domperidone or placebo. The placebo was manufactured to be indistinguishable of domperidone in physical properties. The labelling and packaging were conducted in accordance with Good Manufacturing Practice (GMP), Good Clinical Practice regulations and local or national regulatory requirements. The statistician was blinded to the study group during the study analysis.

Patients received domperidone three daily doses of 10 mg (30 mg/day) for 7 days, plus standard of care (SOC) treatment or matching placebo. A box containing two blisters with 12 tablets were provided to each patient. Since the efficacious daily dose of the active product with viral load reduction capacity was unknown, the maximum labelled dose of the marketed product (10 mg three times daily equal to 30 mg/day for 7 days) was analysed. Dose increasement was not allowed. Labelling and packing of domperidone and placebo followed the GMP regulations and local or national regulatory requirements.

The SOC for SARS-CoV-2 infection included acetaminophen 500 mg (1–4 times daily), non-steroidal anti-inflammatory drugs, symptomatic treatment and hydration for mild COVID-19. In moderate disease only in case of suspicion of bacterial coinfection/superinfection, the following should be prescribed: amoxicillin-clavulanate 875–125 mg every 8 h for 7 days; or alternatively, levofloxacin 500 mg every 12 h on the first day and 500 mg every 24 h for 4 days. Other treatments when required included bronchodilators or inhaled corticosteroids in patients with asthma or chronic obstructive pulmonary disease. Low doses of systemic corticosteroids in patients requiring oxygen therapy and antithrombotic prophylaxis in patients immobilized or with risk factors for thrombosis were allowed [26,27].

### 2.4. Study procedures

In the screening visit (baseline), the eligibility criteria were confirmed, a complete medical history was taken, a SARS-CoV-2 rapid antigen test was performed, a salivary sample was collected for a SARS-CoV-2 PCR test, a peripheral fasting blood sample was drawn for laboratory analyses, the informed consent was signed, and a diary and the study medication were provided. Patients were instructed on how to take the study drug and to complete the diary card, in which the hospitalization criteria were described in plain language.

Telephone contacts were completed at days 1, 4, 7 and 14 after starting treatment. At the end of the study on day 28, patients were visited at the primary care centre. Saliva samples for SARS-CoV-2 PCR assay were collected on baseline at the screening visit. On days 4, 7 and 14 samples were collected from patients’ homes due to limitation of medical visits for quarantined patients. In all telephone contacts, pulse oximetry data, heart rate and temperature recorded were obtained by the patient with the study material supplied for that purpose and were registered by the physician. Questions about the appearance of new symptoms and the severity of previous and actual symptoms were assessed on a numerical rating (NRS) severity scale of 0–10 points (0 = no symptoms, 10 = the most severe symptoms imaginable). Symptoms recorded in the diary card as well as non-prescribed concomitant drugs were communicated to the physician during the telephone calls. In addition, the investigator asked the patients if they have experienced any adverse events since the last study contact, and if any exist, recorded them on the ‘Adverse Event’ case report form page and described the event. All adverse events were followed until their resolution, or chronicity.

All treatments received by the patient, their dose and duration of administration were recorded in the concomitant medications section of the case report form. Throughout the study, site staff maintained adequate records of study drug dispensed, used and returned. At the end of each patient’s participation in the study, all unused medication supplies were accounted by the study monitor to check the treatment compliance.

### 2.5. Viral load

On baseline and days 4, 7 and 14 after the initiation of the treatment, the viral load was determined by detection of three highly conserved epitope regions within SARS-CoV-2 pathogenic viral RNA strain, open reading frame (ORF) 1ab (ORF1ab), nucleocapsid N protein (N Protein) and spike S protein (S Protein), in saliva samples. These analyses were performed in a central laboratory (Arquimea Medical S.L., Leganés, Madrid, Spain). Viral RNA was obtained using the Chemagic™ Viral DNA/RNA 300 kit H96 from (PerkinElmer España, S.L., Tres Cantos, Madrid, Spain), and purification was done using the automated chemagic 360 Instrument (PerkinElmer). RT-qPCR was completed with the TagPath™ COVID-19 CE-IVD RT-PCR Kit (Thermo Fisher, Waltham, MA, USA), and detection of OFR1ab, N Protein and S Protein was completed in the 7500 Real-Time PCR Instrument (Thermo Fisher) and QuantStudio Real-Time PCR Instrument (Thermo Fisher). The sensitivity and specificity of the platform is >99% and 99.5%, respectively. The viral load was estimated as the number of amplification cycles (cycle thresholds, Ct) to detect genes encoding ORF1ab, N Protein and S Protein in a single PCR reaction. An RT-qPCR for SARS-CoV-2 was considered positive in the presence of a Ct value lower than 35 for at least two of the three genes analysed. A higher number of cycles means a lower viral load. Viral load was defined as ‘high’ for Ct values ≤25, ‘medium’ for Ct values >25 and ≤30 and ‘low’ for Ct values ≥30.

### 2.6. Definitions

Asymptomatic or pre-symptomatic infection was defined in the presence of a positive diagnostic RT-qPCR test for SARS-CoV-2 in a patient without symptoms of COVID-19 disease. ‘Mild’ disease was defined in the presence of a positive RT-qPCR test for SARS-CoV-2 in a patient with any COVID-19-related symptoms (e.g. fever, cough, sore throat, malaise, headache, body/muscle pain, nausea/vomiting, diarrhoea, loss of taste or smell) in the absence of tachypnoea, shortness of breath or abnormal findings on chest X-rays. ‘Moderate’ disease was defined in the presence of a positive RT-qPCR test for SARS-CoV-2 in a patient with evidence of lower respiratory tract disease as shown at physical examination (tachypnoea, shortness of breath) or abnormal findings on chest X-rays, with an oxygen saturation (SpO2) level of ≥94% measured by a pulse oximeter. Clinical improvement was defined as a reduction of two or more points in the 0–10 NRS of severity of symptoms [25,26].

### 2.7. Efficacy endpoints

The primary efficacy endpoint was the relative reduction in viral load (day 4 vs. baseline) in each gene (ORF1ab, N Protein, S Protein) in the active treatment group (domperidone plus SOC) as compared with the control group (placebo plus SOC). Secondary efficacy endpoints were the reduction in viral load (day 7 vs. baseline and day 14 vs. baseline); the proportion of patients with a negative RT-qPCR test for SARS-CoV-2 (Ct value >35 in at least two of three genes); the time to achieve a negative viral load from baseline; and the comparison of the clinical efficacy in the two study arms, including reduction in the severity of each symptom (0–10 NRS score) at days 4, 7, 14 and 28 as compared with baseline; proportion of patients with clinical improvement and time to clinical improvement; proportion of patients with disappearance of each symptom at days 4, 7, 14 and 28, and time to disappearance; proportion of asymptomatic patients at days 4, 7, 14 and 28; proportion of patients requiring medical care, admission to the hospital, oxygen therapy and development of complications related to COVID-19 disease over the study period; 28-day mortality rate; mortality rate after the end of study; and safety of domperidone or placebo.

### 2.8. Statistical analysis

The null hypothesis was established as the absence of differences in the reduction of viral load after 4 days of starting treatment as compared with baseline (prior to treatment) between the two study groups. The sample size calculation for the primary efficacy endpoint was performed for a two-sided analysis of variance (ANOVA), with fixed effects and two levels in the factor evaluated corresponding to the active treatment or the control group. A type I error was set at a two-sided 0.05 level with a minimal effect with clinical relevance of 2 log 0 reduction in viral copy number as the minimal difference between the on-treatment groups. A moderate effect of 0.25 (Cohen’s *f*) was targeted leading to an expected common standard deviation (SD) of 4 log 10. Given a sample of 200 patients (100 assigned to domperidone plus SOC and 100 assigned to SOC alone), a power of 94% was obtained to demonstrate the estimated difference (Sample Power, IBM-SPSS; IBM Corp., Armonk, NY, USA). The final sample of 180 patients got a power of 90%. The modified intention to treat (ITT) dataset (all randomized patients who received at least one dose of the study medication) was considered for efficacy and safety analysis.

The main analysis of the primary efficacy endpoint was measured by the Student’s *t* test for independent samples. Imputation rules for missing data were applied to the main analysis, were the mean value at day 4 of the Ct value for each gene was recorded. For the quality control of the study analysis, a sensitivity analysis was applied on the primary efficacy endpoint data and in the evolution of the viral load without imputation rules applied to the dataset. The ANOVA for repeated measurements and a factor (split-plot) with Bonferroni adjustment for multiple comparisons was applied to the comparison of viral load between the study groups at baseline, day 4, day 7 and day 14. The assumptions of the Student’s *t* test and ANOVA were tested, needing the logarithmic transformation of the data for the main analysis to get linear distribution. Kaplan–Meier survival analysis and log-rank test was applied for the analysis of time to get negative PCR. Fisher exact test or Chi^2^ test were applied for the comparison of qualitative variables, and Student’s *t* test for comparison of quantitative variables between study groups. Type I error was established at a two-sided 0.05 level. The software IBM-SPSS 27.0 (IBM Corp., Armonk, NY, USA) was used for the statistical analysis.

## 3. Results

### 3.1. Disposition of patients

**Figure 1.**
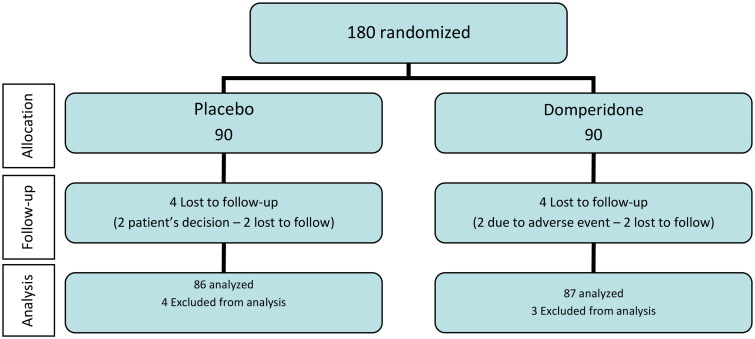
Flow chart of the study population. Analysis was based on the ITT dataset.

A total of 180 patients were recruited by 17 participating centres and were randomized (90 to domperidone and 90 to placebo). Four patients in the placebo group and three patients in the domperidone group were excluded, two of them due to negative RT-qPCR test for SARS-CoV-2 at baseline and the remaining did not take the study treatment. At follow-up, eight patients (four in each study group) withdrew from the study because of patient’s own decision in two, four lost to follow-up and two due to adverse events (abdominal discomfort and dyspepsia). The final evaluable ITT population included 173 patients, 87 in the domperidone group and 86 in the placebo group. The flow chart of the study population is shown in Figure 1.

### 3.2. Clinical characteristics

There were included in the study 109 women (63%) and 64 men (37%). The mean age was 47.8 (SD 1.2) years. The race of the patients was Caucasian in89% and 9.8% Hispanic. A proportion of 15.6% (27 patients) suffered previous COVID-19 with a mean time elapsed to enrolment in the study of 14.2 (1.7) months. The vaccine against SARS-CoV-2 was administered to 159 patients (91.9%) with a mean of 6.3 (0.3) months from the last vaccination dose to study entry. Differences between domperidone and placebo groups in demographics, BMI and previous SARS-CoV-2 infection data were not found (Table 1). No differences were observed in comorbidities frequency between groups.

**Table 1.** Demographic, baseline physical exam and previous SARS-CoV-2 infection in the study groups.

Variables	Total patients (*n* = 173)		Placebo (*n* = 86)		Domperidone (*n* = 87)		*p* value
	*N* (%)	Mean (SD)	*N* (%)	Mean (SD)	*N* (%)	Mean (SD)	
Gender							
Male	64 (37)		33 (38.4)		31 (35.6)		0.709
Female	109 (63)		53 (61.6)		56 (64.4)		
Age (years)		47.8 (1.2)		47.7 (1.7)		47.8 (1.6)	
Race							
Caucasian	154 (89)		74 (86)		80 (92)		0.431
Black	1 (0.6)		1 (1.2)		0		
Hispanic	17 (9.8)		10 (11.6)		7 (8)		
Asiatic	1 (0.6)		1 (1.2)		0		
Body mass index (kg/m^2^)		26 (0.4)		26.6 (0.6)		25.5 (0.5)	0.323
Systolic BP (mmHg)		124.6 (1)		124.4 (1.5)		124.7 (1.3)	0.890
Diastolic BP (mmHg)		76.7 (0.8)		76.4 (1.1)		77 (1)	0.719
Respiratory rate (breaths/min)		15.9 (0.4)		15.6 (0.4)		16.3 (0.8)	0.389
Oxygen saturation (%)		97.2 (0.1)		97.1 (0.1)		97.3 (0.1)	0.318
Heart rate (beats/min)		81.8 (1)		83.1 (1.5)		80.6 (1.3)	0.306
Axillary temperature (°x)		36.4 (0.1)		36.5 (0.1)		36.4 (0.1)	0.788
Previous COVID-19 infection							
No	146 (84.4)		73 (84.9)		73 (83.9)		0.860
Yes	27 (15.6)		13 (15.1)		14 (16.1)		
Severity of previous COVID-19 infection							
Asymptomatic	1 (3.7)		1 (7.7)		0		
Mild	17 (63)		10 (76.9)		7 (50)		
Moderate	8 (29.6)		2 (15.4)		6 (42.9)		
Severe	1 (3.7)		0		1 (7.1)		
Time from previous SARS-CoV-2 infection (months)		14.2 (1.7)		12.6 (2.6)		15.8 (2.1)	0.342

SD: standard deviation.

In relation to severity of COVID-19, 166 patients (96%) presented with mild disease, 4 (2.4%) with moderate disease and 3 (1.7%) were asymptomatic. The distribution of patients according to severity of disease was similar in the two study groups, being in the domperidone and placebo group, respectively, 3 (3.4%) and 0 (0%) no asymptomatic patients, 82 (94.3%) and 84 (99.7%) patients with mild disease, and 2 (2.3%) and 2 (2.3%) patients with moderate disease.

Results of physical examination were similar in the two study groups (Table 1). With a mean of 7.3 (0.3) symptoms, the number of symptoms ranged between 0 and 20. The severity of symptoms was similar in the two study groups (Table 2), although dysgeusia was more frequent in the domperidone group (12 patients, 2.3%) than in placebo (3 patients, 0.6%). Otalgia was significantly more severe in the domperidone alone group (mean 10 points [0.001] vs. 6.7 points [0.6], *p =* 0.004).

**Table 2.** Symptoms observed at baseline: frequency and severity (0–10 points of a NRS) compared by study group.

Symptom	Study group	*N* Placebo (*n* = 87) Domperidone (*n* = 86)	Percentage of patients presenting the symptom (%)	Symptom severity mean (SD)	*p* value for symptom severity comparison
Fever	Placebo	41	47.7	–	
	Domperidone	41	47.1	–	
Cough	Placebo	67	77.9	5.30 (2.30)	0.705
	Domperidone	68	78.2	5.44 (2.08)	
Odynophagia	Placebo	53	61.6	5.70 (2.30)	0.645
	Domperidone	50	57.5	5.48 (2.48)	
Dyspnoea	Placebo	7	8.1	3.71 (2.29)	0.353
	Domperidone	4	4.6	5.00 (1.63)	
Chest pain	Placebo	8	9.3	5.75 (2.87)	0.562
	Domperidone	13	14.9	6.39 (2.06)	
Chills	Placebo	17	19.8	5.65 (2.03)	0.574
	Domperidone	17	19.5	5.24 (2.19)	
Nausea	Placebo	11	12.8	4.27 (2.53)	0.157
	Domperidone	13	14.9	5.77 (2.45)	
Vomiting	Placebo	3	3.5	5.00 (4.36)	0.615
	Domperidone	2	2.3	3.00 (2.83)	
Diarrhoea	Placebo	11	12.8	5.91 (2.43)	0.563
	Domperidone	10	11.5	5.20 (3.08)	
Abdominal pain	Placebo	2	2.3	5.00 (4.24)	0.637
	Domperidone	5	5.8	6.20 (2.39)	
Nasal congestion	Placebo	54	62.8	6.06 (2.24)	0.095
	Domperidone	50	57.5	6.76 (2.02)	
Anosmia	Placebo	6	7	7.17 (2.93)	0.903
	Domperidone	9	10.3	7.33 (2.29)	
Dysgeusia	Placebo	3	3.5	6.33 (3.06)	0.963
	Domperidone	12	13.8	6.42 (2.68)	
Headache	Placebo	55	64	6.35 (2.15)	0.466
	Domperidone	54	62.1	6.04 (2.25)	
Myalgia	Placebo	39	45.4	6.77 (2.06)	0.996
	Domperidone	35	40.2	6.77 (2.17)	
Arthralgia	Placebo	13	15.1	7.00 (2.20)	0.191
	Domperidone	20	23	5.85 (2.54)	
Weariness	Placebo	51	59.3	6.98 (2.06)	0.167
	Domperidone	53	60.9	6.34 (2.59)	
Weakness	Placebo	22	25.6	6.55 (1.84)	0.781
	Domperidone	24	27.6	6.38 (2.24)	
Anorexia	Placebo	18	20.9	6.67 (2.09)	0.087
	Domperidone	16	18.4	7.81 (1.64)	
Dizziness	Placebo	9	10.5	5.44 (2.56)	0.775
	Domperidone	6	6.9	5.83 (2.48)	
Vertigo	Placebo	0	0	–	–
	Domperidone	1	1.2	3.00	
Anxiety	Placebo	1	1.2	6.00	–
	Domperidone	0	0	–	
Insomnia	Placebo	3	3.5	6.00 (1.73)	0.768
	Domperidone	3	3.5	7.00 (5.20)	
Facial pain	Placebo	1	1.2	10.00	0.317
	Domperidone	3	3.5	7.67 (1.53)	
Dysphonia	Placebo	5	5.8	5.00 (2.00)	0.581
	Domperidone	10	11.5	5.70 (2.36)	
Otalgia	Placebo	3	3.5	6.67 (0.58)	0.004
	Domperidone	2	2.3	10.00 (0.01)	
Dry eye	Placebo	1	1.2	7.00	–
	Domperidone	0	0	–	
Blurred vision	Placebo	1	1.2	6.00	–
	Domperidone	1	1.2	5.00	
Foreign body sensation	Placebo	2	2.3	6.00 (1.41)	–
	Domperidone	0	0	–	
Conjunctival congestion	Placebo	1	1.2	9.00	0.454
	Domperidone	2	2.30	7.00 (1.41)	

A total of 102 patients (59%) were receiving treatment for medical conditions at inclusion in the study. No significant differences between the study groups were observed in the administration of concomitant medications (*p =* 0.184).

Compliance of the study treatment was 97% with no significant differences between the study groups.

The viral load was homogeneous between the study groups, with a mean (SD) Ct value of 21.3 (0.4) for ORF1ab, 21.5 (0.4) for N Protein and 27.5 (0.6) for S Protein. The percentages of patients with low, medium and high viral loads were 3.4%, 20.7% and 75.9% for the domperidone group and 4.7%, 14% and 81.4% for the placebo group.

### 3.3. Efficacy endpoints

**Figure 2.**
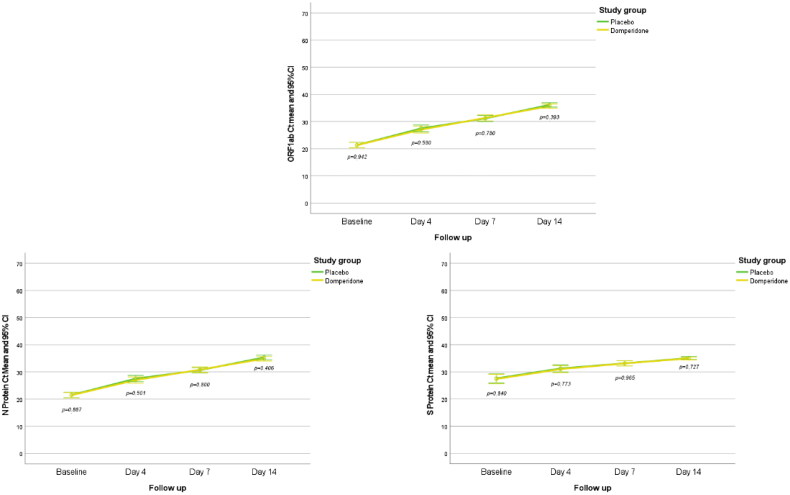
Evolution of Ct values of ORF1ab, N Protein and S Protein at follow-up in the two study groups.

The main study variable was to evaluate changes in viral load from baseline to day 4 and was observed similar in the domperidone and placebo groups for the three SARS-CoV-2 genes, ORF1ab, N Protein and S Protein (Figure 2). For ORF1ab the mean Ct values (SD) were 5.7 (4.9) in the domperidone group as compared with 6.14 (4.8) in the placebo group (mean difference 0.44, 95% CI −1.01 to 1.90; *p =* 0.551). For N Protein, the mean Ct values were 5.56 (4.74) in the domperidone plus SOC group and 6.02 (4.39) in the placebo group, with a mean difference of 0.46 (95% CI −0.91 to 1.83; *p =* 0.505). The mean Ct values for the S Protein were 3.63 (4.60) and 3.65 (5.38) for the domperidone and placebo groups, respectively, with a mean difference of 0.02 (95% CI −1.48 to 1.52; *p =* 0.981).

Ct values of ORF1ab, N Protein and S Protein increased significantly from baseline to days 4 and 14 (*p* < 0.001) in all the patients. There were no significant differences in the ORF1ab Ct values between patients treated with domperidone or placebo at day 4 (*p =* 0.580), day 7 (*p =* 0.780) and day 14 (*p =* 0.393). In addition, no differences were observed for N Protein Ct values at day 4 (*p =* 0.501), day 7 (*p =* 0.800) and day 14 (*p =* 0.406), neither for Ct values of S Protein at day 4 (*p =* 0.773), day 7 (*p =* 0.965) and day 14 (*p =* 0.727; Figure 2).

No significant differences were observed between the main analysis and the sensitivity analysis results.

**Figure 3.**
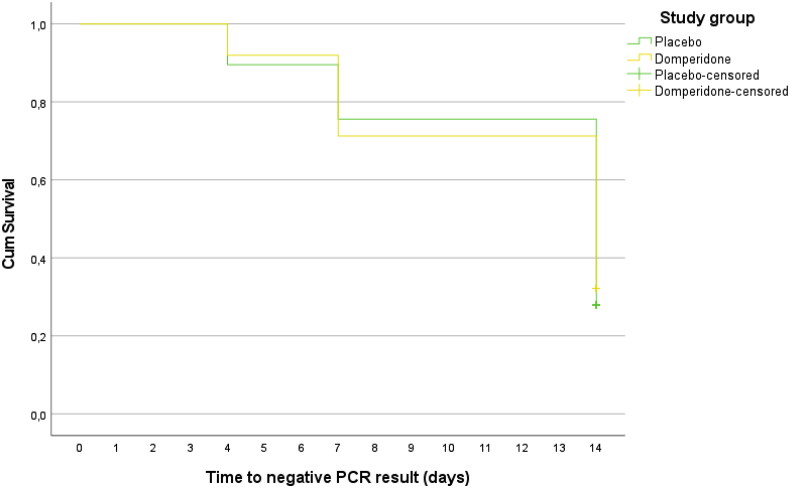
Kaplan–Meier curves for the median time to obtain an RT-qPCR negative result by study group.

No significant differences were found between domperidone and placebo in the proportion of patients with RT-qPCR positive on day 4 (92% vs. 89.5%, *p =* 0.583), day 7 (72.4% vs. 76.7%, *p =* 0.513) or day 14 (32.2% vs. 27.9%, *p =* 0.540). No differences were found between domperidone and placebo in the percentages of patients with low, medium and high viral loads at 4, 7 and 14 days. Globally, 121 patients (69.9%) were negative at day 14, and the viral load was low in 38 patients (22%), medium in 12 patients (6.9%) and high in 2 patients (1.2%). The Kaplan Meier median time to obtain an RT-qPCR negative result was 14 days (95% CI 12.9 to 15.1), without differences between the domperidone and placebo groups (*p =* 0.821; Figure 3).

The vital signs significantly improved from day 1 to day 28 (*p* < 0.01) in the oxygen saturation, heart rate (*p* < 0.01) and axillary temperature (*p* < 0.001) without differences between domperidone and placebo groups. Additionally, there were no significant differences in the severity of the symptoms observed throughout the study. The persistence of symptoms after the end of the study at day 28 was observed in 33 patients (19.1%), without differences between the domperidone or placebo groups (Table 3).

**Table 3.** Persistent symptoms observed after 28 days of follow-up by study groups.

Symptom	Total (*n* = 173)	Placebo (*n* = 86)	Domperidone (*n* = 87)	*p* value
	*N* (%)	*N* (%)	*N* (%)	
Fever	1 (1.6)	1 (3.8)	0	–
Cough	18 (29)	6 (23.1)	12 (33.3)	0.157
Odynophagia	2 (3.2)	0	2 (5.6)	–
Dyspnoea	1 (1.6)	1 (3.8)	0	–
Chest pain	1 (1.6)	0	1 (2.8)	–
Nausea	1 (1.6)	0	1 (2.8)	–
Nasal congestion	4 (6.5)	1 (3.8)	3 (8.3)	0.317
Anosmia	3 (4.8)	1 (3.8)	2 (5.6)	0.564
Dysgeusia	1 (1.6)	1 (3.8)	0	–
Headache	3 (4.8)	2 (7.7)	1 (2.8)	0.564
Myalgia	3 (4.8)	1 (3.8)	2 (5.6)	0.564
Arthralgia	2 (3.2)	2 (7.7)	0	–
Weariness	13 (21)	6 (23.1)	7 (19.4)	0.782
Weakness	3 (4.8)	1 (3.8)	2 (5.6)	0.564
Anorexia	4 (6.5)	1 (3.8)	3 (8.3)	0.317
Anxiety	1 (1.6)	1 (3.8)	0	–
Blurred vision	1 (1.6)	1 (3.8)	0	–

%: Percentage of persistent symptoms in each group.

### 3.4. Safety outcome

Twenty-three patients (13.3%) experienced adverse events, 14 patients in the domperidone group (16.1%) and 9 patients in the placebo group (10.5%), with no statistically significant difference (*p =* 0.276). The total number of adverse events observed was 28, 85.7% (*n* = 24) mild, 10.7% (*n* = 3) moderate. One not related severe palpitation (3.6%) was observed but considered by the physician as not related to domperidone.

Six adverse events were considered possibly related to domperidone (digestive discomfort, dizziness, dyspepsia, epigastralgia, tongue pruritus and tachycardia) and nine unknowns (asthenia, cholelithiasis, increased blood pressure, herpes infection, urticaria, oxygen saturation decrease, nausea, palpitations, paraesthesia, polytraumatism, presyncope, skin rash and vaginitis), with 13 adverse events not related to the study treatment.

Eight adverse events in the placebo group (dizziness, tongue pruritus, nausea, asthenia, paraesthesia, presyncope, epigastralgia, tachycardia) and two adverse events in the domperidone group (abdominal discomfort and dyspepsia) lead to discontinuation of the study drug (*p* = 0.008). No treatment-emergent laboratory abnormalities were observed.

No patients needed to be hospitalized due to the COVID-19 disease, neither needing oxygen therapy. None of the patients died at 28 days after completion of the study.

## 4. Discussion

This clinical trial explored the antiviral activity in the clinical practice of an already marketed product, domperidone, as drug repositioning for COVID-19 treatment. As main result, no significant differences were observed in the evolution of the viral load nor in the symptoms frequency or severity comparing domperidone versus placebo in the 28 days of follow-up.

It has been reported that COVID-19 vaccine can effectively reduce the mortality of COVID-19 infected patients [28] however, studies have shown that even vaccinated, people are infected with new virus variants and immunocompromised patients may not be fully protected after vaccination [29]. In fact, 91.9% of the patients included in our study received previous complete vaccination, a mean of 6.3 months before the study. Although it is unknown the virus variant in the patients included in the study, we know that the SARS-CoV-2 virus variant most frequent at the time of study completion in Spain was Omicron (100%) BA.5 and derivatives [30]. The rate of re-infected patients included in the study was of 15.6% (Table 1) a mean of 14.2 months from first COVID-19 infection. Due to these facts, and in combination to effective vaccination programs, new treatments need to be investigated for moderate-to-severe COVID-19 patients [31], and there is still a need to find treatments against SARS-CoV-2 infection to be administered in the primary outpatient care setting, where most patients of mild-to-moderate disease are attended. They represent more than 70% of COVID-19 patients and can be the first clinical manifestation of perhaps later severe disease. These treatments need to be affordable and easy to use, and oral route should be preferred.

Recently, three oral drugs have shown effective results in clinical trials for COVID-19: molnupiravir, fluvoxamine and Paxlovid. A meta-analysis of the published data [32] showed that the three drugs are effective in reducing the mortality and hospitalization rates in patients with COVID-19 without increasing the occurrence of adverse events. They have potential to be a promising treatment for COVID-19. In recent systematic reviews numerous treatments for COVID-19 are on investigation also with no conclusive results up to date [33,34].

Our group investigated the efficacy of bromhexine as repurposing treatment for COVID-19 compared to SOC group in an open-label phase III clinical trial. The study design and primary care setting were common with the trial presented in this report to facilitate the results comparisons, except the double-blind design that was feasible in the present study that avoided bias in the concomitant medications use. The conclusion was that bromhexine did not offer advantages in the treatment of patients with mild-to-moderate COVID-19 [35]. Relating the drug investigated in this study, no previous clinical trials with domperidone in patients with COVID-19 have been carried out in Spain, and to our knowledge, there is no evidence of the efficacy of this drug in the literature. From the data explained in the introduction to this article it follows that, in addition to its slightly antiviral activity, [1,2] the controlled increase in blood prolactin levels produced by domperidone as dopamine antagonist could stimulate innate and adaptive immunity and increase the survival of patients in certain critical situations, among which can be found patients infected with SARS-CoV-2 [3].

In this randomized double-blind clinical trial, domperidone was compared with placebo. A total of 173 patients were included, being 63% women, ant the mean age of the patients was of 47.8 years. This figure is consistent with global data recorded in Spain for patients with COVID-19, resulting the patients in the age range of 50–69 years the most affected during the pandemic, 55% of them were women [36]. We observed women to be most likely to accept to participate in the clinical trial, but we cannot demonstrate it as gender, or causes for no participation were not recorded. The eligibility criteria for the study limited the recruitment rate, as excluded those patients at higher risk of developing COVID-19. We found some difficulties for the recruitment of patients also due to drastic decrease of COVID-19 in Spain during the study period (March–November 2022). Most patients had mild disease but accounted for a high mean number of clinical symptoms of 7.3 at study entry.

It was remarkable the wide range of symptoms, observing up to 20 symptoms in one patient, and the already known variety of system organ class related (Table 2). The most frequent symptoms affected up to 78% (cough), 63% (headache), 60% (nasal congestion), 60% (odynophagia, weariness) and 47% (fever). About 19% showed persistent symptoms at day 28 with cough, weariness, nasal congestion and anorexia as the most frequent persistent symptoms, and typical COVID-19 symptoms like anosmia, dysgeusia, headache, myalgia and weariness continued in 4.8% (Table 3). It was not explored the effect of the symptoms on the quality of life due to time constraints.

Overall baseline data, including vital signs and symptom distribution, were similar in the two study groups, making them comparable (Tables 1 and 2).

Viral load at baseline was similar in the two study groups as well as the percentages of patients with low, medium and high viral loads. We observe great proportion of patient with high viral load, up to 75.9% for the domperidone group and 81.4% for the placebo group. It was notable that at day 14, 30% of the patients were still positive, although the viral load was low (22%), or medium (6.9%), but still high in 1.2%. We observed the lack of differences between the study groups in the evolution of vital signs, overall improvement of severity of symptoms and percentage of patients with persistent symptoms after day 28.

It was not possible to translate from Ct values to number of viral copies, in consequence the viral loads comparison was not feasible. Regarding the primary efficacy endpoint of as was the reduction in the viral load from baseline-day 4, no differences were found between the domperidone versus placebo patient’s groups for the specific genes of the SARS-CoV-2 pathogenic viral RNA strain. Statistically significant reductions in viral loads were found from baseline for the total study population, but without differences between the study groups. Moreover, the percentage of patients with positive RT-qPCR results at days 4, 7 and 14 and the proportion of patients classified by viral load degree were similar in the two study groups.

In terms of safety outcome, few patients reported adverse events, and there were no significant differences between the study groups. Almost all adverse events were mild and not related to treatment. Eight adverse events in the placebo group and two adverse events in the domperidone group lead to discontinuation of the study drug but no patients needed to be hospitalized due to the COVID-19 disease.

The double-blind design is a strength of the study. The effect of domperidone on the evolution of symptoms could not be properly evaluated due to the limited sample size and the wide range of symptoms observed in patients with COVID-19, but no differences were observed. The increasement of prolactine levels occur with the doses of domperidone used in the study assuring the effects described in the introduction but could not be measured for this trial. We observed limitations due to distant communication and difficulty interpreting the symptoms severity by the patients and physicians. In addition, the study size did not allow the evaluation of the effect on hospitalization rates nor mortality, considering that the patients included in the study with mild-to-moderate COVID-19 had lower hospitalization and mortality risk. The inclusion of 96% patients with mild disease where almost all cure spontaneously limited the observation of risk events. With the inclusion of more moderate cases perhaps differences between treatment groups might be observed. It is unknown if the effect of domperidone could be different as per virus variant.

## 5. Conclusion

The viral load reduction of ORF1ab, N Protein and S Protein genes at day 4, with the treatment with domperidone or placebo, was not significantly different as resulted in this clinical trial. From the results of this study, no information for the recommendation of the use of domperidone as antiviral for treating patients with mild-to-moderate COVID-19 was obtained.

Further research is needed to explore whether a higher dose of domperidone than used in this study might provide the expected antiviral action and also needs more investigation if the administration of the drug for more than 7 days might improve the clinical results, or if the use as preventive might avoid the disease instauration.

## Data Availability

The datasets used and analysed during the current study are available from the corresponding authors upon reasonable request.
